# Cryoablation with drug-loaded bead embolization in the treatment of unresectable hepatocellular carcinoma: safety and efficacy analysis

**DOI:** 10.18632/oncotarget.24029

**Published:** 2018-01-08

**Authors:** Jian-Ying Zeng, Xiang-Hao Piao, Zhong-Yuan Zou, Qing-Feng Yang, Zi-Lin Qin, Ji-Bing Chen, Liang Zhou, Li-Zhi Niu, Jian-Guo Liu

**Affiliations:** ^1^ Central Laboratory, Affiliated Fuda Cancer Hospital, Jinan University, Guangzhou 510665, P.R. China; ^2^ Department of Intervention, Affiliated Fuda Cancer Hospital, Jinan University, Guangzhou 510665, P.R. China; ^3^ School of Medicine, Jinan University, Guangzhou 510632, P.R. China; ^4^ Department of Surgical Oncology, Affiliated Fuda Cancer Hospital, Jinan University, Guangzhou 510665, P.R. China

**Keywords:** hepatocellular carcinoma, transarterial chemoembolization, cryoablation, hepasphere microsphere, doxorubicin

## Abstract

This study aimed to explore the efficacy and safety of drug-eluting bead (DEB) embolization (DEB-TACE) when combined with cryoablation in the treatment of unresectable hepatocellular carcinoma (HCC). The study was a single-center randomized controlled trial comprised of 60 patients with HCC conducted between August 2015 and October 2017. The patients were randomly divided into two groups: DEB-TACE combined with cryoablation (DEB-TACE-Cryo group) or cryoablation alone (Cryo group). Inter-group differences in overall survival, progression-free survival, and adverse reactions were assessed. The operative success rates were 82.7% and 77.4% in the DEB-TACE-Cryo group and Cryo group, respectively, with no operative mortality. The overall survival and progression-free survival in the DEB-TACE-Cryo group were significantly higher than those in the Cryo group (16.8 months vs.13.4 months, *P* = 0.0493; 8.1 months vs. 6.0 months, *P* = 0.0089, respectively). The postoperative complications in the two groups were rated as grade 1 or grade 2, according to guidelines set by the National Cancer Institute Common Terminology Criteria for Adverse Events Version 4.0 (CTCAE V4.0). We demonstrated that DEB-TACE combined with cryoablation was effective, well tolerated, and had a low complication rate. Therefore, this combination therapy may be a better choice for the treatment of unresectable hepatocellular carcinoma.

## INTRODUCTION

Worldwide, hepatocellular carcinoma (HCC) is the second leading cause of cancer death in men and the sixth in women. During 2012, an estimated 782,500 new HCC cancer cases were diagnosed; However, due to very poor prognosis, the number of deaths was almost the same (745,500) [1]. For very early stage and early stage patients, surgical resection, radiofrequency ablation (RFA), and/or liver transplantation are effective options for treatment [2]. Unfortunately, nearly 30% of patients who have been diagnosed at intermediate or advanced stages have no surgical options [3–4]. Most patients diagnosed with unresectable HCC succumb to liver failure as a result of advancing cirrhosis or tumor progression [5]. Transarterial chemoembolization (TACE) and sorafenib are recommended palliative therapies for patients with unresectable HCC [2]. However, the efficacy of TACE depends on the size of the tumor. For tumors larger than 5 cm in diameter, the rate of complete necrosis is low and the long-term outcome is negatively affected [6]. Accordingly, TACE combined with other therapies, such as local liver ablation, has been widely applied and proven to significantly improve tumor response and patient survival rates [7–11]. Cryoablation is another alternative local treatment option for unresectable HCC. Compared to RFA, cryoablation has several unique advantages including more clearly visualized margins of ablated lesions from normal tissue on ultrasound (USG) or computed tomography (CT), larger range of ablation zones, less pain and tumor immunosuppressive effect [12–16]. It has been demonstrated that cryoablation combined with conventional TACE treatment can further improve on the therapeutic effect of cryoablation alone against HCC [17–21]. However, the safety and efficacy of TACE with drug-eluting beads (DEBs) combined with cryoablation for HCC have not yet been reported. DEBs are a relatively novel drug delivery embolization system that facilitate anticancer agent release and fixed dosing [22–25]. HepaSphere microspheres, commercially available DEBs, are biocompatible, hydrophilic, non-resorbable, expandable, and conformable microspheres designed for controlled, targeted embolization. They have been shown to be highly effective in the treatment of HCC [26–29].

With the above in mind, this randomized controlled clinical trial combined cryoablation with DEB-TACE, using 50–100 μm diameter HepaSphere microspheres for the treatment of unresectable HCC. The aim of this study was to assess the overall survival, progression-free survival, and adverse events after combined therapy, with a view of achieving a safer and more effective treatment method for unresectable HCC.

## RESULTS

### Clinical data

From August 2015 to September 2016, 397 patients received liver tumor treatment in our hospital. 290 cases were diagnosed as HCC. In accordance with the eligibility criteria and exclusion criteria, 73 patients with portal vein embolization, 59 patients with extrahepatic multiple metastases, 34 patients with obvious hepatic artery-portal vein fistulas or artery-venous fistulas, 8 patients with coagulation dysfunction, and 24 patients with severe liver dysfunction were excluded. Additionally, 32 people refused to participate in the study. Ultimately, there were 60 patients with HCC who met the inclusion criteria and agreed to study enrollment. The clinical characteristics for the 60 patients are listed in Table 1.

**Table 1:** Demographics and tumor parameters of study participants

Characteristic	DEB-TACE-Cryo group (n = 29)	Cryo group (n = 31)	*P*
**Sex**			
F	3 (10.3)	5 (16.1)	0.5101
M	26 (89.6)	26 (83.9)	
**AJCC stage (2010)**			
IIIA	14 (48.3)	20 (64.5)	0.7189
IIIB	8 (27.6)	6 (19.4)	
IIIC	4 (13.8)	3 (9.7)	
IVA	3 (10.3)	3 (9.7)	
**Viral marker**			
HBsAg positive	18 (62.1)	20 (64.5)	0.9202
Anti-HCV positive	2 (6.9)	2 (6.5)	
**Child-Pugh stage**			
A	28 (83.3)	30 (90.3)	0.9617
B	1 (16.7)	1 (9.7)	
Mean age (years)	65.3 ± 12.7	56.5 ± 11.0	0.0513
**ECOG score**			
0	11 (37.9)	13 (41.9)	0.2626
1	12 (41.4)	16 (51.6)	
2	6 (20.7)	2 (6.5)	
Lesion size (cm)	7.2 ± 4.5	6.5 ± 3.8	0.6310
**AFP, IU/mL**			
< 200	18	16	0.4860
200 – 400	3	2	
> 400	8	13	
ALT, U/L	29.3 ± 14.2	38.9 ± 13.5	0.0960
AST, U/L	50.7 ± 21.9	42.1 ± 23.0	0.2642
TBIL, μmol/L	14.9 ± 6.8	19.3 ± 9.2	0.1691
**ALB, g/L**	33.9 ± 3.4	37.6 ± 4.3	0.0507
28–35	12	6	0.0628
> 35	17	25	
**Tumor type**			
Single massive	17 (58.6)	25 (80.6)	0.0628
Multinodular	12 (41.4)	6 (19.4)	
**Ascites**			
Yes	8	7	0.6545
No	21	24	
**Cancer pain**			
Yes	5(17.2)	2 (6.4)	0.1933
No	24 (82.8)	29 (93.6)	

### Treatment success rate and tumor response

**Figure 1:**
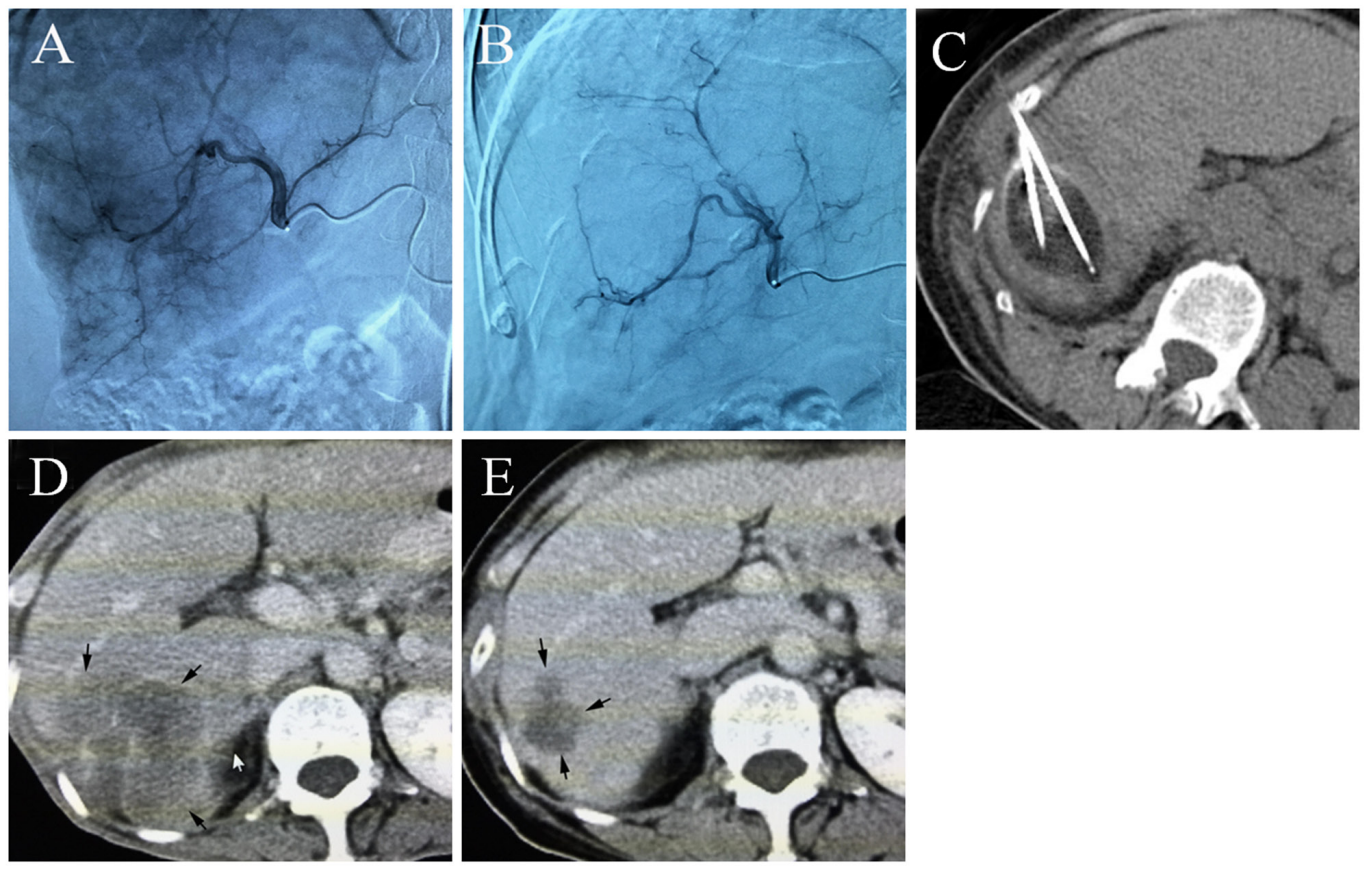
**(A)** Common artery angiography of a 65 year old male patient with HCC, selective catheterization of the pathologic branch of the right hepatic artery supplying the tumor, **(B)** the artery angiography shows that most of the tumor staining disappeared after DEB-TACE, **(C)** percutaneous cryoablation under CT guidance, an ice ball was formed, **(D)** the contrast-enhanced CT scan shows a huge tumor before treatment, **(E)** five months after DEB-TACE-Cryo treatment, the enhanced CT showed that the tumor was significantly reduced.

Four to six weeks after treatment, 5 patients and 7 patients with residual active tumors were identified in the DEB-TACE-Cryo group and Cryo group, and the intervention success rates were 82.7% (24/29) and 77.4% (24/31), respectively. All 8 patients with residual active tumors underwent a second cryoablation cycle (Figure 1). Six months and 1 year after treatment, there were 5 and 11 cases with local recurrence or distant metastasis in the DEB-TACE-Cryo group, respectively. This recurrence rate represents 17.2% and 37.9% (7 local recurrence and 9 distant metastasis), respectively. In the Cryo group, there were 7 cases and 21 cases with local recurrences or metastases, and the recurrence rates were 22.6% and 67.7% (8 local recurrences, 13 distant metastases), respectively. The difference in the recurrence rate of the two groups one year after treatment was statistically significant (*P* = 0.0207).

### Survival analysis

At the time of censor, 14 and 21 patients had expired in the DEB-TACE-Cryo group and Cryo group (*P* = 0.1264), respectively. The median follow-up was 17.2 ± 5.5 months (range, 6 to 26.4 months).

**Figure 2:**
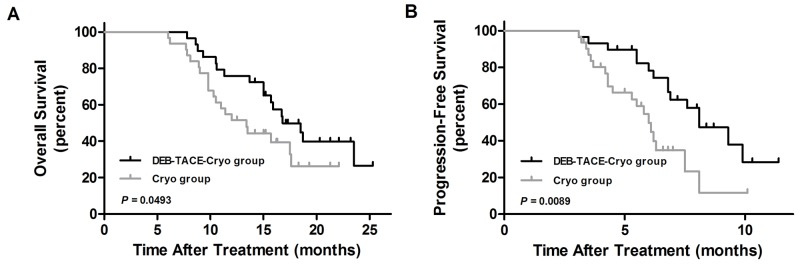
The overall survival curves **(A)** and progression-free survival curves **(B)** of transarterial chemoembolization with drug-eluting beads combined with cryoablation (DEB-TACE-Cryo) or cryoablation (Cryo) for unresectable hepatocellular carcinoma (HCC).

The 0.5, 1, and 2 year overall survival rates in the DEB-TACE-Cryo group and Cryo group were 100%, 89.7%, and 62.1%, and 100%, 83.9%, and 41.9%, respectively (Figure 2A). The median overall survival in the DEB-TACE-Cryo group (16.8 months) was longer than that in the Cryo group (13.4 months), the difference was statistically significant (Hazard Ratio = 0.557; 95% Cl, 0.284 to 1.093; *P* = 0.0493).

The 0.5, and 1 year progression-free survival percentages for the DEB-TACE-Cryo group and the Cryo group were 82.8% and 62.1%, and 77.4% and 32.3%, respectively (Figure 2B). The median progression-free survival in the DEB-TACE-Cryo group (8.1 months) was longer than that in the Cryo group (6.0 months), the difference was statistically significant (Hazard Ratio = 0.367; 95% Cl, 0.175 to 0.771; *P* = 0.0089).

The causes of death for 8 patients were tumor progression for seven, liver failure for two, and another disease for one of the DEB-TACE-Cryo group. For the Cryo group, the causes of death for 13 patients were tumor progression for nine, liver failure for two, and other diseases for two (*P* = 0.7292, *P* = 0.9450, and *P* = 0.5937, respectively).

### Complications

**Figure 3:**
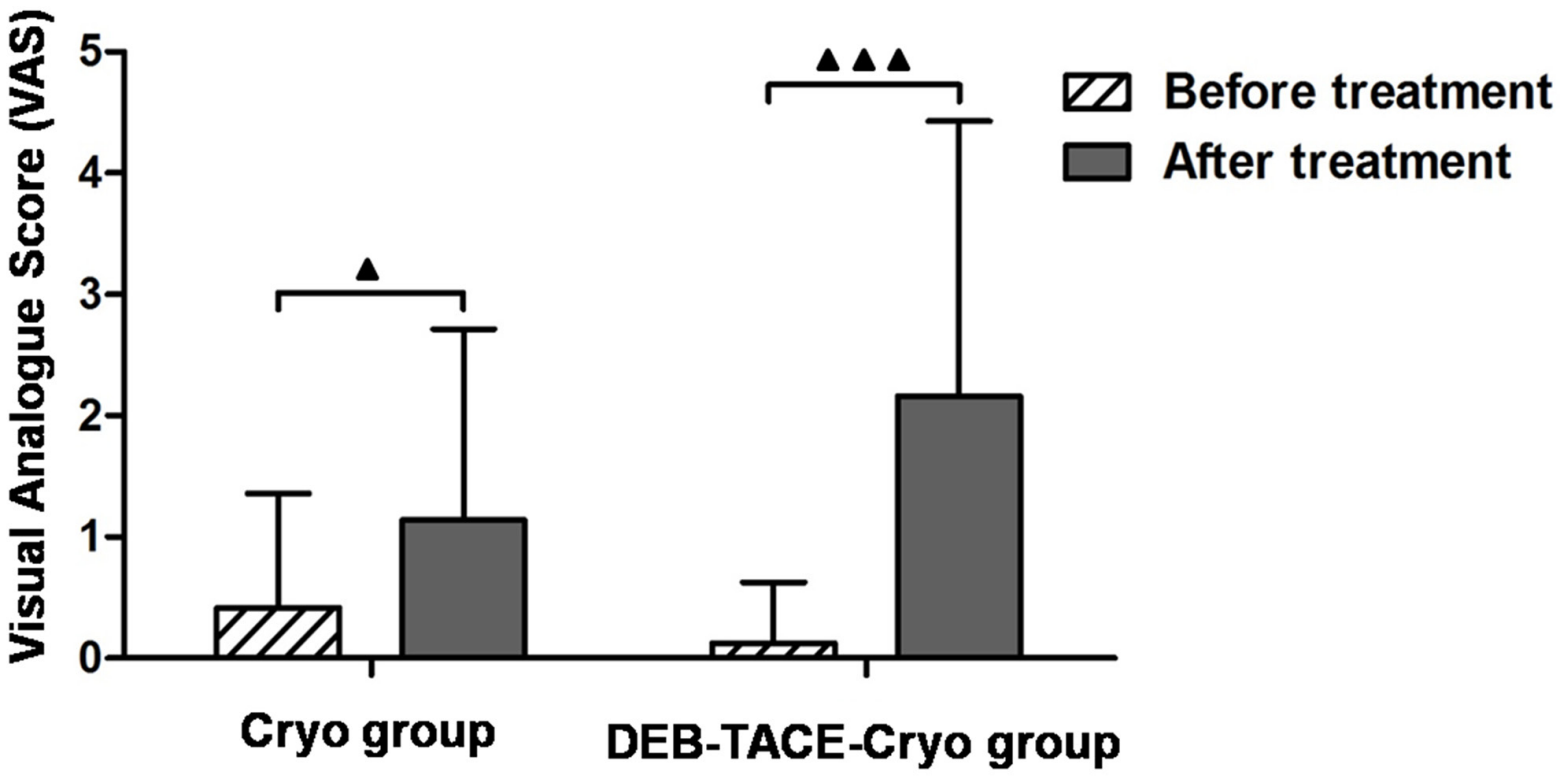
Preoperative and postoperative pain scores in patients with unresectable hepatocellular carcinoma (HCC) treated with transarterial chemoembolization using drug-eluting beads combined with cryoablation (DEB-TACE-Cryo) or cryoablation (Cryo)

There was a 0% 30 day mortality rate. The common complications in the two groups were as follows: fever; pain; coughing; ascites; nausea; pleural effusion; thrombocytopenia; erythropenia; and elevated blood pressure. The most common complication was pain in both groups. The maximum pain scores in the two groups after treatment were significantly higher than those before treatment (*P* = 0.01 and *P* < 0.000, Figure 3). There was no significant difference in incidences of complications between the two groups (*P* > 0.05, Table 2). According to CTCAE V4.0, the postoperative complications in the two groups were rated as grade 1 or grade 2.

**Table 2:** Rates of the most common complications after treatment

Group	DEB-TACE-Cryo group	Cryo group	*P*
Fever	6 (20.7%)	2 (6.5%)	0.1398
Pain	21 (72.4%)	17 (54.8%)	0.1881
Cough	5 (17.2%)	3 (9.7%)	0.4653
Ascites	3 (10.3%)	5 (16.1%)	0.5101
Nausea	1 (3.4%)	1 (3.2%)	0.5144
Pleural effusion	5 (17.2%)	5 (16.1%)	0.5101
Thrombocytopenia	3 (10.3%)	5 (16.1%)	0.5101
Erythropenia	3 (10.3%)	1 (3.2%)	0.2693
Elevated blood pressure	3 (10.3%)	4 (12.9%)	0.7577

### Postoperative liver function changes

**Figure 4:**
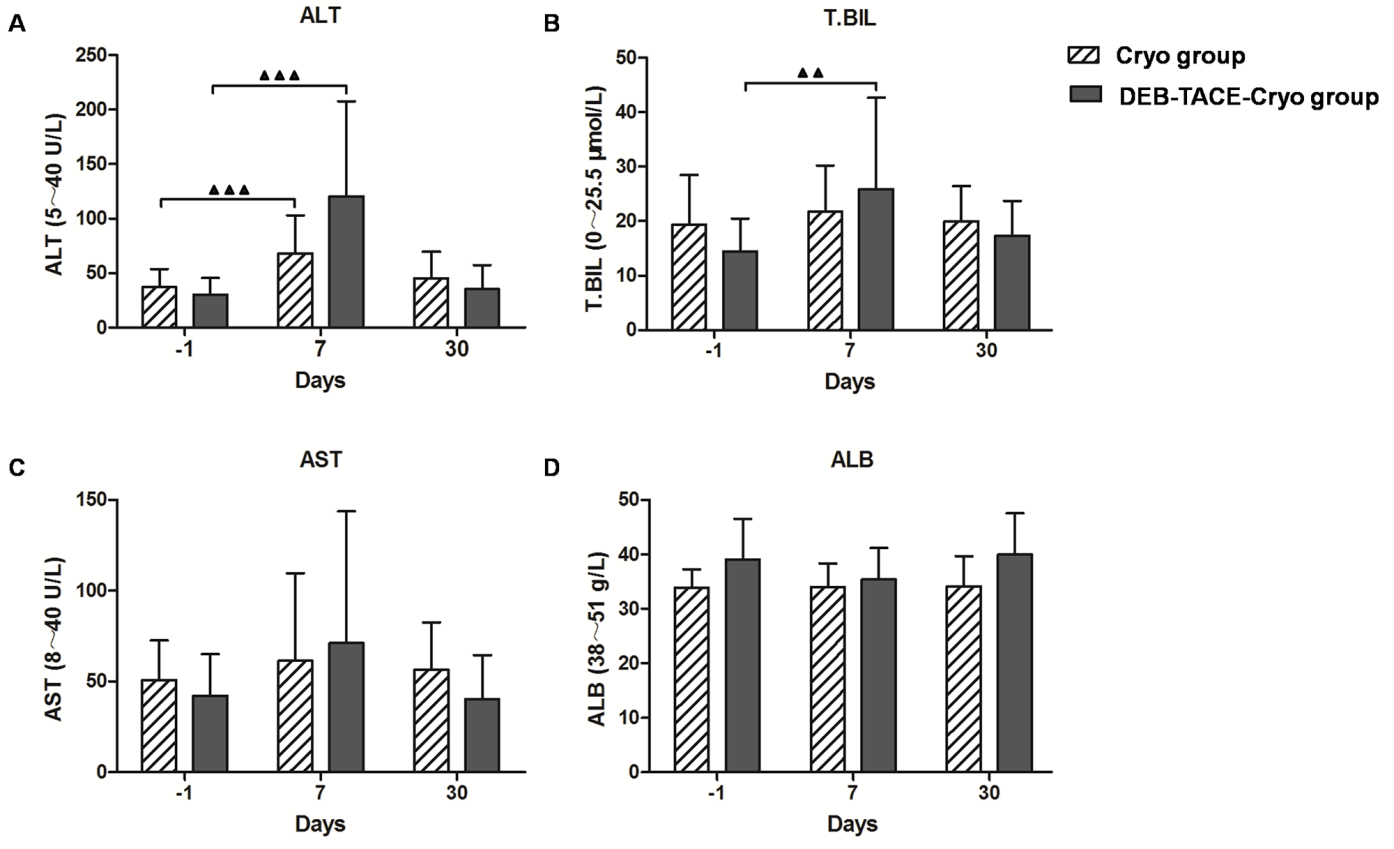
Changes in hepatic functional reserve before and after treatment in both groups In the DEB-TACE-Cryo group, the serum ALT and TBIL levels were 29.3 ± 14.2 U/L and 14.9 ± 6.77 μmol/L, respectively. Seven days after treatment, the ALT and TBIL values increased significantly (to 114.6 ± 92.1 U/L and 24.0 ± 15.4 μmol/L, respectively, *P* = 0.002 and *P* = 0.016; **A and B**). In the Cryo group, the ALT levels were 38.9 ± 13.5 U/L. Seven days after treatment, the ALT values increased significantly (to 73.2 ± 30.1 U/L, *P* = 0.008; A). No obvious change in AST or ALB levels were observed after treatment (*P* > 0.05; **C and D**).

Seven days after treatment, the serum alanine aminotransferase (ALT) levels of both the DEB-TACE-Cryo and the Cryo groups and serum total bilirubin (T.BIL) in the DEB-TACE-Cryo group had increased during the short-term (*P* = 0.008, *P* = 0.002, and *P* = 0.016, respectively). Interestingly, there was no significant increase recorded for any of these levels 30 days after treatment. No obvious changes in aspartate aminotransferase (AST) or albumin (ALB) levels were observed after treatment (Figure 4).

## DISCUSSION

According to follow-up results, both the treatment success rate and short-term tumor response in the DEB-TACE-Cryo group were higher than the Cryo group.The DEB-TACE-Cryo group also achieved a longer progression-free survival and overall survival than the Cryo Group (*P* < 0.05). Therefore, this study showed that combined therapy with DEB-TACE and cryoablation resulted in better treatment efficacy than single therapy with cryoablation for patients with unresectable HCC.

The combined therapy for DEB-TACE-Cryo group during this trial period resulted in a success rate of 82.7% (24/29) within 4–6 weeks, and 1 and 2 year survival rates of 89.7% and 62.1%. Veltri et al. reported similar efficacy when RFA combined with TACE was applied for the treatment of unresectable HCC, with survival rates of 89.7% after 12 months and 67.1% after 24 months of treatment [30]. Using a similar RFA combinatory approach, Zhang et al. reported that complete ablation was achieved in 76.2% of cases, with 1, 2, and 3 year survival rates of 89%, 61%, and 43%, respectively [31]. Similarly, Zhao et al. reported a 1 year survival rate of 88.5% [32]. Considering these comparative results, we believe DEB-TACE-Cryo treatment for HCC may be considered as an effective palliative treatment option for patients with unresectable HCC.

There are several reasons for performing DEB-TACE before cryoablation. Firstly, DEB-TACE embolizes tumor internal blood vessels and reduces the heat-drop effect when the ice ball meets the blood vessel, thus promoting the rate of tumor necrosis. Secondly, the local ablation rate is related to the size of the tumor[19, 33]. As DEB-TACE can cause tumor ischemia thus reducing the tumor oxygen supply and release of chemotherapeutic drugs from microspheres in a sustained fashion over a prolonged period of time, it can effectively inactivate the tumor [34–35], which reduces the tumor volume, and increases the rate of complete ablation through cryoablation. Thirdly, DEB-TACE before cryoablation controls intrahepatic micro-lesions, which inhibit recurrences after treatment.

From a safety aspect, the postoperative complications of the two groups were classified as grade 1 or 2 according to the CTCAE V4.0 standard, neither of which is considered to be very severe. There was no significant difference in the incidence of complications between the two groups(*P* > 0.05), which showed that combination therapy did not cause the superposition effects or increases incidence rate of complication. Notably, according to the liver function results in this study, the serum ALT levels in the two groups and the T.BIL in the DEB-TACE-Cryo group increased during the first 7 days. However, there was no significant increase recorded 30 days after treatment. This indicates that neither treatment approach resulted in long-term liver function effects and that both the combined therapy and cryoablation alone are safe for unresectable HCC treatment.

In conclusion, DEB-TACE using HepaSphere microspheres combined with cryoablation may be a more effective approach to improve the outcome for patients with unresectable HCC than cryoablation alone. Use of DEB-TACE using HepaSphere microspheres combined with cryoablation could represent a valuable therapeutic option in unresectable HCC patients. However, since this study is a single-center, small sample randomized clinical trial, we believe multicenter and large-scale clinical trials are warranted to increase the objective accuracy of data in the future.

## MATERIALS AND METHODS

### Ethics

The study protocol received ethical approval from the Regional Ethics Committee of the Guangzhou Fuda Cancer Hospital, China. Written informed consent was obtained from each participant, in accordance with the Declaration of Helsinki.

### Patient selection

This prospective randomized controlled study was registered at clinicaltrials.gov [identification number NCT02545556] and conducted in the central laboratory of The Affiliated Fuda Cancer Hospital, Jinan University, Guangzhou, China from August 2015 to October 2017.

According to eligibility and inclusion criteria, 60 patients were enrolled in the study and randomly divided into two groups using a stratified randomization algorithm: the DEB-TACE-Cryo group (29 patients), treated with HepaSphere drug loaded microsphere embolization combined with cryoablation; and the Cryo group (31 patients), subjected to cryoablation alone. Randomization was stratified according to two variables: sex and tumor staging.

For all patients, the diagnosis of HCC was based on the diagnostic criteria used by the European Association for the Study of the Liver [36]: (1) pathological diagnostic criteria: the biopsy specimens were confirmed by histopathological examination (n = 43); (2) clinical diagnostic criteria: with cirrhosis or hepatitis B virus (HBV) and/or hepatitis C virus (HCV) infection, the characteristics of typical HCC imaging and/or serum α-fetoprotein (AFP) ≥ 400 IU/mL for 1 month or ≥ 200 IU/mL ug/L for 2 months (n = 17).

The eligibility criteria were as follows: (1) Eastern Cooperative Oncology Group (ECOG) score ≤ 2; (2) platelet count ≥ 100 × 10^9^/L; (3) white blood cell count ≥ 3 × 10^9^/L; (4) neutrophil count ≥ 2 × 10^9^/L; (5) hemoglobin ≥ 90 g/L; (6) prothrombin time international normalized ratio ≥ 1.5; (7) hepatic tumor not obviously invading the gallbladder, diaphragm or large vessels; (8) absence of level 3 hypertension, severe coronary disease, myelosuppression, respiratory disease, and acute or chronic infection; (9) Child-Pugh score A or B; (10) renal function (serum creatinine < 130 μM, serum urea < 10 mM).

The exclusion criteria were (1) Child-Pugh score C; (2) the main portal vein was completely embolized by tumor thrombus, and lack of collateral vessels; (3) multiple distant metastases, estimated survival time of less than three months; (4) proportion of the total liver tumor was more than 70% cancer; (5) obvious hepatic artery-portal vein fistula, or artery-venous fistula.

### TACE with HepaSphere

#### Preparation of HepaSphere microspheres

Preparation was performed as suggested by the manufacturer. Each vial of 50–100 μm HepaSpheres (HepaSphere™, South Jordan, Utah, USA) was loaded with 25 mg of doxorubicin diluted in 20 mL of 0.9% NaCl solution for injection. The 20 mL of doxorubicin solution was aspirated into two separate 20 mL syringes. One of the 20 mL syringes containing 10 mL of doxorubicin was added to the HepaSphere vial and agitated frequently for 10 min, after which the remaining 10 mL were added. The vial was agitated every 10–15 min for 60 min to complete the ionic bonding of doxorubicin. After the loading period, all the supernatant was extracted, and an equal quantity of nonionic contrast diluted with saline (50:50) was added to form the final suspension for injection. Overall, the final injectable volume from each vial was 30 mL. Before beginning to inject the suspension, 100 μm of glyceryl trinitrate (nitrolingual) was injected through the microcatheter at the target vessel to achieve the maximum vessel dilatation, and the microcatheter was flushed with hyperheparinized saline (2.500 IU/500 mL of flushing saline). Slow, incremental injection of the suspension followed at a rate of 1–3 mL/min until obliteration of the neovascularity. When initial stasis had been achieved, a further pause of 3–5 min was allowed to facilitate the redistribution of the spheres within the lesion and the directed distal push of the spheres by blood inflow pressure. After this pause, more embolic material was injected under the same flow conditions. The total number of vials delivered at each session was recorded.

### Catheterization

The interventional procedure was performed in a dedicated interventional angiography suite and followed the classical steps of hepatic artery catheterization and embolization. After administration of local anesthesia in the right groin, a 5F sheath was introduced into the right common femoral artery. Selective catheterization of the celiac trunk and superior mesenteric artery was followed by superselective catheterization of the right and left hepatic arteries. Depending on the randomization, HepaSphere microspheres loaded with doxorubicin were administered by a transcatheter technique under fluoroscopic guidance. After administration, a second hepatic angiography was performed to look for a residual vascular tumor blush.

### Cryoablation

Each procedure comprised two freeze-thaw cycles accomplished using an argon gas-based cryosurgical unit (Endocare, Irvine, CA, USA). Cryoprobes (3 mm in diameter) were inserted into the center of the tumor mass under ultrasonographic guidance, each reaching a temperature of -150 °C at the tip of the probe. The duration of freezing depended on the achievement of an ice ball that extended 1 cm beyond the boundary of the tumor and was visible as a hypoechoic region on ultrasonography. Generally, the maximum freezing time was 15 min, followed by thawing for 5 min; this cycle was then repeated. For masses < 6 cm in long diameter, two or three cryoprobes were placed within the center of the tumor, to ensure freezing of the entire mass. For masses with a long diameter of 6–10 cm, the tumor was divided into two parts that were treated in turn, usually with an interval of 1 week. For masses with a long diameter of > 10 cm, the tumor was divided into three parts treated at intervals of usually 1 week. The tracts formed were sealed with fibrin glue immediately after removal of the cryoprobes to ensure hemostasis.

In the DEB-TACE-Cryo group, percutaneous cryoablation was conducted under the guidance of USG and/or CT within the three weeks after DEB-TACE (median, 9 days; range, 3–18).

### Follow-up and response assessment

To assess the success of the technique, contrast-enhanced CT or magnetic resonance imaging (MRI) were performed 4–6 weeks after treatment. The efficacy evaluation was based on the modified response evaluation criteria in solid tumors (mRECIST). The imaging evaluation of the treatment result was made using a double blind method. Two radiologists, each with more than ten years of experience, independently interpreted the radiographic findings and consensus was reached on collective reading if their opinions were divided. If the outcome was considered a complete response, the treatment was considered successful and then follow up was conducted every three months. If residual active tumor remained, a second cryoablation cycle was performed after re-evaluation of the patient.

At each visit, HCC patients underwent radiographic and hematologic examinations. If distant multiple metastases were found, a symptomatic treatment or other palliative therapy was applied.

### Safety

Enhanced CT and bedside ultrasonography were performed within 24 h of the ablation. The purpose of this first follow-up imaging was primarily to check for complications and not to evaluate treatment efficacy. Additionally, in order to evaluate the effect of the intervention on liver function, serum ALB, AST, ALT, and T.BIL levels were measured 7 and 30 days post-procedure. Complications within 30 days of treatment were evaluated in accordance with the criteria established by the Society of Interventional Radiology (SIR) and were graded according to the National Cancer Institute Common Terminology Criteria for Adverse Events Version 4.0 (CTCAE V4.0). Preoperative and postoperative pain scores were taken according to the Visual Analogue Score (VAS) criteria.

This study was censored on October 9, 2017.

### Statistical analysis

All statistical analyses and all associated figures were performed and compiled using GraphPad software (GraphPad, San Diego, CA, USA). The continuous data, categorical data, and survival curves of the two groups were compared using the Mann Whitney, Chi-square, and Gehan-Breslow-Wilcoxon tests individually. Continuous data from the same group were compared using the paired *t* test. The results were expressed as the mean ± standard deviation. All statistical tests were two-sided and a *P* value < 0.05 was considered statistically significant.

## Abbreviations

DEB: drug-eluting beads
TACE: transarterial chemoembolization
Cryo: cryoablation
HBsAg: hepatitis B surface ntigen
HBV: hepatitis B virus
HCV: hepatitis C virus
AJCC: American Joint Committee on Cancer
ECOG: Eastern Cooperative Oncology Group
AFP: α-fetoprotein
ALT: alanine aminotransferase
AST: aspartate aminotransferase
T.BIL: total bilirubin
ALB: albumin
